# Nasopharyngeal carcinoma presenting as Garcin’s syndrome: A rare case report

**Published:** 2015-10-07

**Authors:** Shivani Patel, Apurva Patel, Monil Majmundar, Irappa Madabhavi, Ravi Shah, Jay Soni

**Affiliations:** 1 Department of Internal Medicine, Civil Hospital, College of B.J. Medical, Gujarat University, Gujarat, India; 2 Gujarat Cancer and Research Institute AND Department of Medical and Pediatric Oncology, College of B.J. Medical, Gujarat University, Gujarat, India

**Keywords:** Nasopharyngeal Carcinoma, Multiple Cranial Neuropathies, Radiotherapy

Garcin syndrome consists of unilateral palsies of almost all cranial nerves without either sensory or motor long-tract disturbances and without intracranial hypertension, and it is caused by a malignant osteoclastic lesion at the skull base.^1^ The underlying cause of Garcin’s syndrome is usually a sarcoma, lymphoma, metastasis, chemodectoma, or carcinoma of the skull base. The literature on Garcin’s syndrome presenting as an early sign of nasopharyngeal carcinoma (NPC) is limited, and this makes the present case rare and interesting.

A 70-year-old man, smoker, presented to our institute with complain of headache, drooping of right eyelid, double vision, facial asymmetry, and dysphagia to solid and liquid, dysarthria, hoarseness of voice for 4 months (Figure 1). He had developed difficulty in hearing for 1 month and episodes of syncope in last 15 days.

General physical examination revealed cervical lymphadenopathy. On central nervous system examination, there was complete ptosis and no ocular movements were possible on the right side. Diplopia was present on all gazes. Pupils were bilaterally equal and reacting normally to light. There was decreased sensation over the right side of the face with absent ipsilateral conjunctival and corneal reflex. There was a facial asymmetry with the loss of forehead wrinkle on the right side. Pure tone audiometry showed a conductive hearing loss in the right ear. Indirect laryngoscopy examination revealed right-sided vocal cord palsy, uvula deviated to left side and gag reflex was also absent. The tongue was atrophied on the right side and on protrusion deviated to the right. Thus, 9 out of 12 cranial nerve were involved in this patient. The rest of the systemic examination was within normal limits. Fundus examination and cerebrospinal fluid examination was within normal limits.

A magnetic resonance imaging of the head and neck region revealed primary nasopharyngeal mass lesion of size 49 × 62 × 47 mm extending inferiorly, obliterating Eustachian tube; laterally to pterygomaxillary fissure, sphenopalatine fossa; cavernous sinus, internal carotid artery; right inferior orbital fissure with perineural invasion through right foramen ovale and foramen rotundum (Figure 2). Histopathological examination of the excision biopsy specimen revealed high-grade malignant tumor cells in syncytial arrangement, with large nucleus, vesicular chromatin, prominent nucleoli, and moderate amount of cytoplasm with indistinct cytoplasmic borders suggesting poorly differentiated primary NPC (Figure 3).

**Figure 1:**
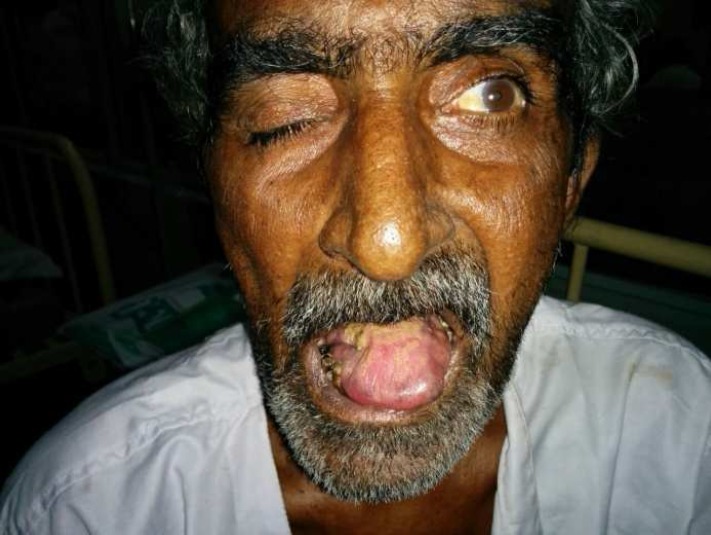
Clinical photograph showing ptosis, loss of nasolabial folds, and deviation of angle of mouth to opposite side

**Figure 2:**
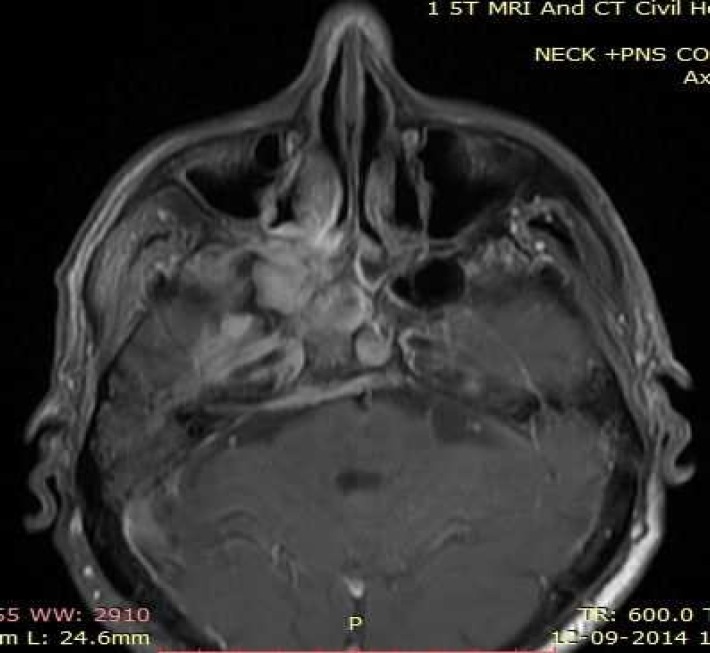
A gadolinium enhanced magnetic resonance imaging of the head and neck region revealed primary nasopharyngeal mass lesion of size 49 × 62 × 47 mm

Thus, the combination of signs, symptoms, radiological and histopathological findings enabled us to diagnose as a case of Garcin’s syndrome secondary to NPC. The patient was managed with two cycles of neo adjuvant chemotherapy (DCF protocol: docetaxel, cisplatin and 5-flurouracil) along with growth factor support which he tolerated very well. Concurrent chemo radiotherapy was given with weekly cisplatin along with radiotherapy at a dose of 1.8 Gy per fraction for 30 fractions. There was a dramatic improvement in his symptoms in the form of improvement in the hoarseness, facial weakness, headache, giddiness, dysphagia, dysarthria and diplopia. Now the patient is under regular surveillance at oncology clinic since 6 months.

**Figure 3:**
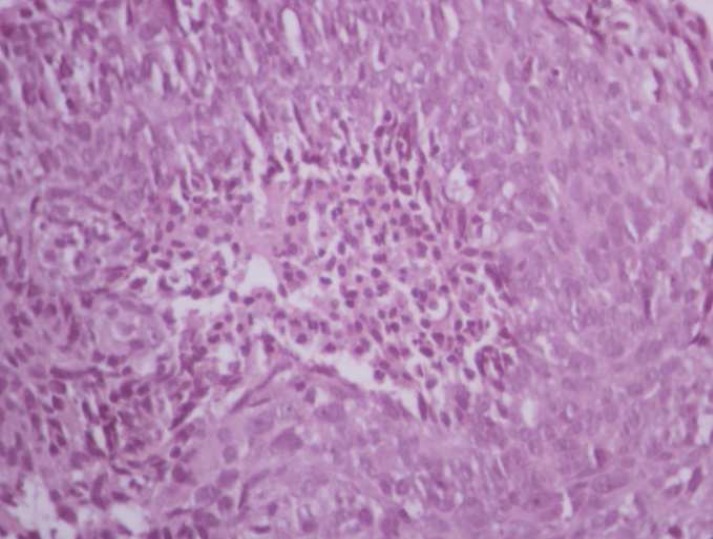
Histopathological examination revealed high grade malignant tumour cells in syncytial arrangement, with large nucleus, vesicular chromatin, prominent nucleoli, and moderate amount of cytoplasm with indistinct cytoplasmic borders suggesting poorly differentiated nasopharyngeal carcinoma

Garcin syndrome is an ipsilateral step-by-step deterioration of all 12 cranial nerves, first described in 1927. This rare progressive condition is generally associated with skull based malignant osteoclastic lesions but has also been described with pachymeningitis secondary to otitis media, rhinocerebral mucormycosis, hypertrophic pachymeningitis, lymphomatous meningitis, carcinomatous leptomeningitis, chemodectoma, meningioma, and giant internal carotid aneurysm. NPC rarely comes to medical attention before it has spread to regional lymph nodes. Enlargement and extension of the tumor in the nasopharynx may result in symptoms of nasal obstruction, changes in hearing, and cranial nerve palsies. The most common physical finding is a neck mass consisting of painless firm lymph node enlargement (80%).

Cranial nerve palsy at initial presentation is observed in 25% of patients. This tumor may involve the cranial nerves in many ways. First, the tumor may extend superiorly through foramen lacerum, which is an unimpeded pathway near the fossa of Rosenmuller into the cranium 3, 4, 5, thus involving the cranial nerves in the middle cranial fossa and cavernous sinus. Cancer may break through the pharyngo-basilar fascia and spread along vascular sheaths, that is, facial planes surrounding the jugular vein and carotid artery. However, NPC solely presenting as Garcin’s syndrome is rarely represented in the literature.^2^ Lateral and posterior extension of the primary growth itself may involve the lower cranial nerves exiting from jugular and hypoglossal foramina. These lower cranial nerves may also be involved on their course while traversing the neck by the secondary deposits in the lymph nodes.

The treatment options for NPC are radiotherapy, chemotherapy and concurrent chemoradiotherapy. Chemoradiation significantly improved progression-free survival and overall survival.^3^

## Notes:

**How to cite this article: **Patel S, Patel A, Majmundar M, Madabhavi I, Shah R, Soni J. Nasopharyngeal carcinoma presenting as Garcin’s syndrome: A rare case report. Iran J Neurol 2015; 14(4): 228-30.
